# Emerging therapeutic drug monitoring technologies: considerations and opportunities in precision medicine

**DOI:** 10.3389/fphar.2024.1348112

**Published:** 2024-03-13

**Authors:** Winnie S. Liang, Brett Beaulieu-Jones, Susan Smalley, Michael Snyder, Laura H. Goetz, Nicholas J. Schork

**Affiliations:** ^1^ Net/Bio Inc, Los Angeles, CA, United States; ^2^ Translational Genomics Research Institute (TGen), Phoenix, AZ, United States; ^3^ University of Chicago, Chicago, IL, United States; ^4^ Stanford University, Stanford, CA, United States

**Keywords:** therapeutic drug monitoring, N-of-1 clinical trials, biosensors, wearables, precision medicine

## Abstract

In recent years, the development of sensor and wearable technologies have led to their increased adoption in clinical and health monitoring settings. One area that is in early, but promising, stages of development is the use of biosensors for therapeutic drug monitoring (TDM). Traditionally, TDM could only be performed in certified laboratories and was used in specific scenarios to optimize drug dosage based on measurement of plasma/blood drug concentrations. Although TDM has been typically pursued in settings involving medications that are challenging to manage, the basic approach is useful for characterizing drug activity. TDM is based on the idea that there is likely a clear relationship between plasma/blood drug concentration (or concentration in other matrices) and clinical efficacy. However, these relationships may vary across individuals and may be affected by genetic factors, comorbidities, lifestyle, and diet. TDM technologies will be valuable for enabling precision medicine strategies to determine the clinical efficacy of drugs in individuals, as well as optimizing personalized dosing, especially since therapeutic windows may vary inter-individually. In this mini-review, we discuss emerging TDM technologies and their applications, and factors that influence TDM including drug interactions, polypharmacy, and supplement use. We also discuss how using TDM within single subject (N-of-1) and aggregated N-of-1 clinical trial designs provides opportunities to better capture drug response and activity at the individual level. Individualized TDM solutions have the potential to help optimize treatment selection and dosing regimens so that the right drug and right dose may be matched to the right person and in the right context.

## Introduction

TDM is the measurement of drug concentration(s) in blood, plasma, or other bio-samples, in order to determine the optimal drug dosing regimen for an individual (Kang and Lee, 2009; Clarke, 2016; Ates et al., 2020). Its adoption has been historically limited due to challenges with available techniques, which include chromatographic strategies that may be coupled with immunoassays or other detection methods (Ates et al., 2020). While these approaches have utility, wider implementation has been hindered due to factors including issues with low throughput and inaccurate detection despite high sensitivity and specificity for chromatography methods; and low specificity, in spite of lower costs, simpler protocols, and high-throughput flexibility, for immunoassay approaches (Carlier et al., 2015; Ates et al., 2020; Tuzimski and Petruczynik, 2020). However, more recent technological developments will enable more widespread TDM applications in the clinic and in research. One area that will benefit from these developments is precision medicine, which holds promise towards better tailoring effective drug treatments to improve the health of patients, and also improving our understanding of drug pharmacokinetics (PK) and pharmacodynamics (PD) at the individual level. Finding solutions to effectively match drugs and doses to patients is needed, particularly due to the fact that although a large variety of drugs are routinely prescribed by physicians or are available as over the counter (OTC) drugs, there have not been improvements in health in the general population for some time (Sánchez-Sánchez et al., 2021); and further, more than 50% of prescribed or dispensed drugs are used inappropriately (Sánchez-Sánchez et al., 2021). In addition, an individual may take multiple drugs to treat different conditions, potentially creating problematic drug-drug interactions, or may take dietary supplements, which are not regulated by or registered with the Food and Drug Administration (FDA), but may be marketed to promote health benefits. New TDM technologies thus have the potential to enhance scientific understanding of which drugs truly benefit an individual’s health.

While TDM has been used in specific contexts, there are opportunities to widen its scope of use. Drugs for which TDM are commonly used include anti-epileptic drugs (Patsalos et al., 2018), antibiotics (Muller et al., 2018; Wicha et al., 2021; Abdul-Aziz et al., 2022), anti-cancer drugs (Decosterd et al., 2015; Buclin et al., 2020), and others (Punyawudho et al., 2016; Imamura, 2019). Key criteria used to determine which drugs may be appropriate for, or benefit from, TDM include those that demonstrate: 1) inter-subject PK variability, 2) intra-subject PK stability over time, 3) a clear correlation between drug concentration and clinical response and/or toxicity, 4) a narrow therapeutic window, 5) in-availability of PD biomarkers of clinical response and/or drug toxicity, and 6) consistent treatment duration to enable dosage changes (Ates et al., 2020; Buclin et al., 2020). Meeting these criteria requires rigorous trials and analyses that are not performed for all prescription and OTC drugs, often due to significant time and cost investments, but could, in theory, be used to optimize the use of any drug. TDM would also benefit from the use of pharmacogenetic testing to improve drug prescription strategies. Such testing was implemented in, for example, the PREPARE (Pre-emptive Pharmacogenomic Testing for Preventing Adverse Drug Reactions) study, which utilized a 12-gene pharmacogenomic panel encompassing 50 germline variants to assess adverse reactions associated with a genotype-guided drug treatment compared to standard of care (Swen et al., 2023). Notably, using genotype-guided drug treatments resulted in a 30% decrease in clinically relevant drug reactions (Swen et al., 2023). Integrating strategies such as pharmacogenetic testing with TDM will be important to consider in order to maximize therapeutic benefits for patients.

Outlining efficient strategies to determine which drugs are suitable for TDM, which subjects would benefit from TDM, and how to appropriately apply TDM in these situations remains a challenge due to the unique comorbidity, genetic, epigenetic, behavioral, and environmental exposure profiles that each individual possesses (Kang and Lee, 2009; Landmark et al., 2016). The use of strategies such as biosensor and wearable technologies, as well as medical digital twins, computational simulations of real-world patients that utilize key features of an individual to forecast how they may respond to injury, infection, or treatments (Laubenbacher et al., 2022) and which have specifically shown promise for personalizing pain medication management (Bahrami et al., 2023), have the potential to address such challenges, alleviate the burden of implementing TDM strategies, and also enable the use of continuous drug monitoring. Continuous monitoring is particularly attractive for facilitating precision medicine as it: 1) creates a closed-loop system for real-time assessment of drug responses and fine tuning of doses; 2) can help to expedite drug development and clinical trials by quickly identifying clinically meaningful trends of a drug’s effects; 3) enables the collection of longitudinal data, *versus* the collection of temporally fragmented data, to improve reliability of predictions and to strengthen data interpretation; and 4) can be used to delineate intra- and inter-individual variability in drug response and PK to ultimately improve individual treatment outcomes, which may be extrapolated to larger populations (Bian et al., 2021). Ideally, such efforts, in combination with pharmacogenetic testing, could decrease the incidence of adverse events (AEs), minimize drug toxicity, improve tolerability, reduce costs, decrease burden on both patient and clinical staff, and improve therapeutic outcomes (Bian et al., 2021). Such benefits may be further maximized using N-of-1 clinical trials, which treat each subject as an independent study, and which may be used to determine if a subject responds to an intervention, and to determine the most effective treatment for that subject (Schork and Goetz, 2017; Selker et al., 2022). Data from separate trials may in turn be aggregated to make broader claims across a population. By taking into account the unique nature of each individual, these designs differ from traditional trials, which are designed to evaluate interventions in the greater population and whose aim is not necessarily to find an effective treatment for each subject (Schork and Goetz, 2017; Selker et al., 2022). TDM also relies on the assumption that a drug’s PK informs its PD, but this does not always hold true (Open Resources for Nursing and Ernstmeyer, 2023). N-of-1 analyses will thus help to characterize inter-individual variability in these associations. The strategic implementation of new TDM technologies within an N-of-1 framework has the potential to advance personalized medicine in novel ways. In this mini-review, we will discuss emerging TDM technologies and key factors that impact TDM, as well as opportunities to implement N-of-1 and aggregated N-of-1 designs, to maximize the benefits of TDM in the conduct of precision medicine.

## Emerging TDM technologies

New technologies potentiating TDM include biosensors and wearables which can enable the translation of specific measurements on individuals into quantifiable drug-induced signals (Ates et al., 2020). Drug-induced signal detection from, e.g., plasma samples, typically occurs as a result of non-covalent binding of a recognition element (antibodies, enzymes such as cytochrome P450 (i.e., enzyme-linked assays (ELA)), membranes, polymers, or aptamers) to an analyte (Ates et al., 2020) and is performed most commonly using optical and electrochemical methods (Garzón et al., 2019; Ates et al., 2020; Pollard et al., 2021; Qian et al., 2021). With optical methods, a biorecognition event generates an optical signal, or elicits a change in environmental optical properties, which is subsequently captured by a photodetector (Dincer et al., 2019; Kim et al., 2019). This approach is used to measure concentrations of antibiotics (Zengin et al., 2014; Cappi et al., 2015; Losoya-Leal et al., 2015; Spiga et al., 2015; Tenaglia et al., 2018), anti-cancer drugs (Zhao et al., 2015; Yockell-Lelièvre et al., 2016), antifungals (Berger et al., 2017), anti-epileptic drugs (Yamada et al., 2015), therapeutic drug antibodies (Lu et al., 2017; Beeg et al., 2019), and others (Liu et al., 2020; Bian et al., 2021; Weber et al., 2021) (Table 1). With electrochemical methods, a biorecognition event generates an electrical signal proportional to the drug concentration (Dincer et al., 2019). Electrochemical biosensors have been used with antibiotics (Kling et al., 2016; Bruch et al., 2017; Yu et al., 2018; Dauphin-Ducharme et al., 2019), anti-epileptics (Mobed et al., 2022), anti-cancer drugs (Tajik et al., 2015; Lima et al., 2018; Sukanya and Rath, 2022), as well as antifungals (Tuchiu et al., 2022) (Table 1). Both optical and electrochemical biosensors demonstrate similar advantages including high sensitivity, reliability, and multiplexing capabilities, with electrochemical solutions also enabling on-site monitoring and usage of small sample volumes (Dincer et al., 2019; Ates et al., 2020). Disadvantages associated with optical biosensors include their susceptibility to background noise and environmental interference, potential signal loss depending on the matrix that is used, the fragility of instrumentation, and high instrumentation costs (Dincer et al., 2019; Ates et al., 2020), while electrochemical approaches may harbor issues with non-specific binding of analytes (Ates et al., 2020). In general, biosensor utility for TDM is affected by factors such as the degree of invasiveness of sample collection for analyte analysis and signal amplification strategies which may increase the sensitivity and the selectivity of signal detection (Dincer et al., 2019; Ates et al., 2020).

**TABLE 1:** TDM biosensor technologies evaluated using human matrices.

Detection method	Biosensor technology	Recognition element(s), nanomaterial(s)	Additional features	Sampling matrix	Monitored drug(s)	References
Electrochemical	Conductive cotton (fiber)-based, ion-selective electrode	Carbon nanotubes, ion-selective membrane cocktail	Miniaturized, flexible, and wearable sensor; continuous monitoring; no preconditioning required	Human plasma	Lithium	Sweilam et al. (2018)
Electrochemical	Cyclic voltammetry (continuous TDM)	Pencil graphite electrode	Raspberry Pi-based circuit board; integration into a fluidics system; data sharing through phone app and smart watch	Undiluted human serum	Propofol, paracetamol	Stradolini et al. (2018)
Electrochemical	Differential pulse voltammetry	Carbon nanotubes	Flexible sensor patch	Sweat	Methylxanthine (caffeine)	Tai et al. (2018)
Electrochemical	Differential pulse voltammetry	Gold nano-dendritic structures	Flexible sensor patch	Sweat	Levodopa (L-dopa)	Tai et al. (2019)
Electrochemical	Enzyme-linked assay	Antibody	Single use, microfluidic lab-on-chip design	Diluted human plasma	ß-lactam antibiotics (piperacillin, cefuroxime, cefazolin)	Bruch et al. (2017)
Electrochemical	Enzyme-linked assay	Antibody	Microfluidic, multi-analyte detection	Human plasma (spiked)	Tetracycline, pristinamycin	Kling et al. (2016)
Electrochemical	Field-effect transistor-based	Thiolated aptamer, 6-mercapto-1-hexanol	Real-time drug monitoring	Human plasma	Tenofovir	Aliakbarinodehi et al. (2017)
Electrochemical	Memristive biosensor	Aptamer, silicon nanowire-arrays		Undiluted human serum	Tenofovir	Tzouvadaki et al. (2017)
Electrochemical	Potentiometric microneedle-based	ß-lactam hydrolysis; Iridium-oxide layer	Minimally-invasive; pH-sensitive iridium oxide coating	Blood, interstitial fluid	ß-lactam antibiotics; penicillin	Gowers et al. (2019)
Electrochemical	Potentiometric microneedle-based	ß-lactam hydrolysis; Iridium-oxide layer	Real-time drug monitoring; pH-sensitive iridium oxide coating	Human blood and extracellular fluid	ß-lactam antibiotic (phenoxymethylpenicillin)	Rawson et al. (2019)
Optical	Microdialysis-supported immunoassay	Antigen-immobilized amino-modified chip; fluorophore-conjugated antibody	Microfluidic, quasi-continuous sampling	Whole human blood; ultracentrifuged human plasma	Cyclosporine, mycophenoloic acid	Weber et al. (2021)
Optical	Surface plasmon resonance	Tumor necrosis factor-immobilized flow cell	Microfluidic (Biacore X100)	Diluted human serum	Infliximab	Grasmeier et al. (2023)
Optical	Surface plasmon resonance	Antigen-immobilized nanoplasmonic chip, gold nanodisks	Competitive immunoassay	Diluted human serum	Acenocoumarol	Peláez et al. (2018)
Optical	Surface plasmon resonance	Antibody-conjugated gold nanoparticles		Diluted human serum	Adalimumab	Bian et al. (2018)
Optical	Surface plasmon resonance, immunoassay	Antibody-conjugated gold nanoparticles		Diluted human serum, plasma, whole blood (spiked), dried blood spots	Infliximab	Lu et al. (2017)
Optical	Surface-enhanced Raman spectroscopy	Inkjet-printed paper gold nanoparticles	Paper-based, passive vertical flow membrane system	Undiluted human serum (spiked)	Flucytosine	Berger et al. (2017)

Additional factors influencing TDM include the sample matrix that is used and how samples are collected. The most commonly used matrices for TDM are plasma and whole blood and thus the relationships between matrix drug concentration and therapeutic response are best characterized for these sample types (Ates et al., 2020). However, variability in hematocrit across subjects may introduce bias in TDM (Ates et al., 2020; Sikma et al., 2020). Thus, use of other types of matrices, such as sweat, interstitial fluid (ISF) and oral fluids, are being explored (Kiang et al., 2012; Ghareeb and Akhlaghi, 2015; Gao et al., 2019) and will enable wider adoption of TDM studies and practices. The mode of sample collection may also influence the success of TDM. Microsampling technologies such as dried blood spots (Gaissmaier et al., 2016; Zakaria et al., 2016; Li et al., 2021) and remote collection alternatives such as those commercially available from Neoteryx (Gruzdys et al., 2019; Williams et al., 2021) or Tasso (Williams et al., 2021; Wan et al., 2022), may help decrease the invasiveness, burden, and cost of sample collection, but will also require testing and validation of reliability. Furthermore, timing of sample collection may introduce an incomplete picture of drug concentration levels, especially for drugs with long half-lives or if the subject has hepatic and/or renal insufficiency affecting drug metabolism (Ates et al., 2020). Solidifying continuous TDM solutions will aid in resolving these issues.

### Continuous TDM solutions

Continuous TDM yields significant benefits over traditional TDM, whereby measurements have been typically collected only at single or specific time points (Hiemke, 2008). In addition to providing a more comprehensive view of drug concentration changes over an period of time, continuous TDM can also improve optimization of therapeutic dosing and treatment decision-making, reduce drug toxicity, enable characterization of PK dynamics within and across subjects to aid in creating more reliable PD and PK models, reduce burden on the subject and on clinical staff, and ultimately help to expedite clinical trials and drug development (Bian et al., 2021). To perform continuous TDM, electrochemical biosensors may be used as they can be modified using functional nanomaterials and immobilized antibodies or aptamers to improve matrix analysis and target capture, respectively; and can be integrated with microfluidic and wearable, or implantable, devices (Bian et al., 2021).

Both *in vitro* and *ex vivo* methods have demonstrated the utility of electrochemical biosensors for continuous TDM. *In vitro* methods include measurements on extracted blood or buffers, whereas *ex vivo* methods involve the use of a discrete substrate outside of the body (Bian et al., 2021). *In vitro* methods encompass approaches that modify electrode surfaces with nanomaterials to improve biosensing capabilities (Maduraiveeran et al., 2018; Bian et al., 2021; Vaneev et al., 2022), and have been used to monitor drugs including naproxen (Baj-Rossi et al., 2014; Stradolini et al., 2018; Sweilam et al., 2018), propofol and paracetamol (Stradolini et al., 2018), and lithium (Sweilam et al., 2018). Additional elements that may be used are aptamers, single-stranded DNA or RNA molecules that have high target binding affinity and specificity (Bian et al., 2021), which have been used for detection of drugs including tenofovir (Aliakbarinodehi et al., 2017; Tzouvadaki et al., 2017), vancomycin (Dauphin-Ducharme et al., 2019), imatinib (Tartaggia et al., 2021) and anti-fungals (Wiedman et al., 2017). In contrast to *in vivo* approaches, *ex vivo* methods utilize an external monitoring substrate such as a microfluidic device, or a wearable sensor (Bian et al., 2021). While microfluidic-based sensors have been primarily tested in animal models to continuously monitor drugs such as doxorubicin (Ferguson et al., 2013; Karnik, 2017; Mage et al., 2017; Bian et al., 2021), promising wearable options that utilize sweat or microneedle sensors are being explored for humans (Gao et al., 2016; Chung et al., 2019; Bian et al., 2021). These types of wearable biosensors can be placed on the epidermis to measure drugs and analytes in sweat, following physical activity or through sweat induction (Tai et al., 2018; Chung et al., 2019; Tai et al., 2019), or from ISF which is accessed from microneedle penetration into the dermal-interstitial space (Goud et al., 2019; Gowers et al., 2019; Rawson et al., 2019). Despite challenges around sufficient sample collection and validating blood *versus* sweat-based drug concentrations, wearable sweat biosensors have been used to perform real-time monitoring of caffeine (Tai et al., 2018) and levodopa (Tai et al., 2019). Microneedle-based sensors have similar challenges, such as the need to improve detection limits, as they capture measurements from ISF. This approach is most commonly used for monitoring plasma glucose for management of diabetes (Lee et al., 2016; Wang et al., 2016), but also has utility for continuous TDM of levodopa (Goud et al., 2019) and antibiotics (Gowers et al., 2019; Rawson et al., 2019).

*In vivo* biosensors, which are suitable for feedback-controlled closed-loop systems, can also be used for continuous TDM and drug administration. These solutions, which are commonly used in the form of implantable biosensors for measuring and maintaining normal plasma glucose levels in diabetic subjects, represent an optimal strategy towards precision drug management as they allow for a more complete view of PK changes within and across subjects (Bian et al., 2021). *In vivo* biosensors outside of glucose monitoring have been primarily explored in animal models to monitor doxorubicin and tobramycin (Arroyo-Currás et al., 2017), and feedback-controlled dosing of vancomycin (Dauphin-Ducharme et al., 2019). One notable observation from animal studies is the high level of inter-animal variance (>50%) in PK-related measurements of drug distribution, excretion, and maximum plasma concentration, and the absence of an association between these factors and body surface area (Vieira et al., 2019). This is further exacerbated by metabolic variation across species (Bian et al., 2021) and emphasizes the need to develop and optimize TDM at the individual level. Overall, improvements in sensor technology, including smart bandages (Mostafalu et al., 2018; Dincer et al., 2019), disposable wearable sweat and ISF sensors (Zhang et al., 2016; Ainla et al., 2018; Kim et al., 2018; Dincer et al., 2019), voltammetry-based sensing modalities that do not rely on recognition elements (Lin et al., 2020), and integration of sensors into smartphone-based tools (Madrid et al., 2022), will pave the way for future adoption of these solutions.

## Considerations and opportunities for TDM in precision medicine

### Polypharmacy, supplement use, and drug/supplement interactions

One key challenge with traditional or continuous TDM is determining how to perform analyses and interpret data in the context of drug combinations, polypharmacy, and the use of dietary supplements. Polypharmacy is the simultaneous use of five or more prescription and non-prescription medications by one person (Masnoon et al., 2017). At least four out of ten older adults meet this definition and almost 20% take at least ten drugs (Brownlee and Garber, 2019). When including dietary supplements and OTCs, approximately 67% of older adults fulfill the definition of polypharmacy (Qato et al., 2016). Polypharmacy can lead to serious drug interactions, decreased adherence to medication (Elbeddini et al., 2021), suboptimal treatment (Darwich et al., 2017), and an increase in the risk of AEs by 7%–10% with each medication that is taken (Elbeddini et al., 2021). Oversight of dietary supplements is particularly challenging, since it is estimated that they are used by 80% of all adults (Levinson, 2012). However, only 23% do so based on the advice of their healthcare professional (Akabas et al., 2016). Furthermore, since quality standardization of supplements is minimal, there are significant safety, quality, and efficacy concerns (Ronis et al., 2018). Based on AEs submitted to the FDA, 40,546 AEs resulting from consumption of vitamins, minerals, proteins, and unconventional diets have been reported since 2004 (FDA, 2023). Although TDM has been primarily applied towards monitoring of prescription drugs, expanding its application to supplements is critical, especially given the possibility of synergistic or antagonistic effects of co-administered medications (Shipkova and Christians, 2019).

**FIGURE 1:**
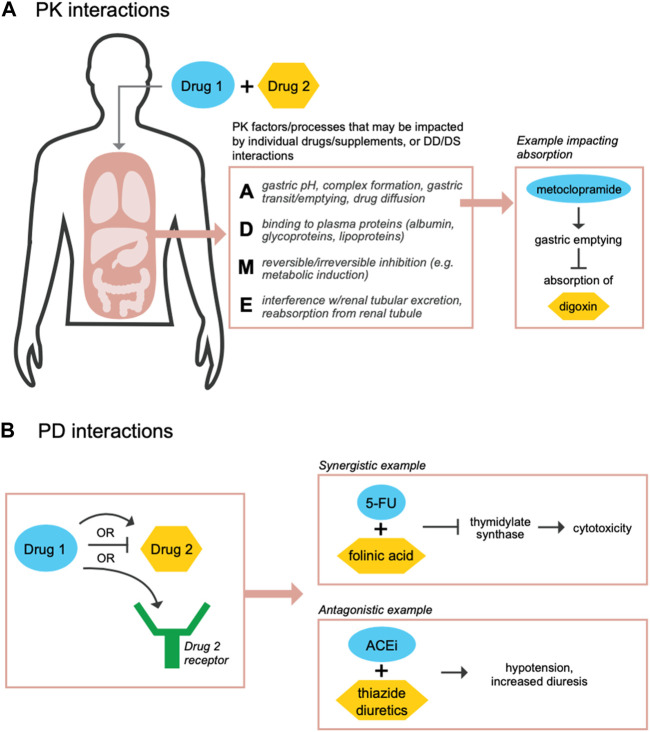
Co-administration of drugs and supplements may result in PK and/or PD-related interactions. During co-administration of drugs or supplements, PK drug-drug (DD) or drug-supplement (DS) interactions may occur if the individual compounds share mechanisms or impact processes across absorption **(A)**, distribution **(D)**, metabolism (M), and/or excretion **(E)** functions **(A)** (Palleria et al., 2013; Grogan and Preuss, 2023). Such interactions may cause in a change in the concentration of the drug or supplement at its site of action. For example, metoclopramide, a dopamine receptor antagonist that treats nausea and vomiting in patients with gastroesophageal reflux disease, may activate gastric motility and decrease absorption of drugs such as digoxin, a heart failure medication (Johnson et al., 1984). Separately, PD, DD or DS, interactions may occur if one of the compounds has a direct effect on the mechanism of the other compound **(B)**. For example, a synergistic interaction occurs when 5-fluorouracil (5-FU) is administered with folinic acid to increase inhibition of thymidylate synthase in order to kill cancer cells (Keyomarsi and Moran, 1986; Niu et al., 2019); alternatively, an antagonistic interaction occurs when angiotensin converting enzyme inhibitors (ACEi) are administered with thiazide diuretics, which are used to treat hypertension, resulting in increased hypotension and diuresis (Mignat and Unger, 1995; Niu et al., 2019).

Since TDM is based on a relationship between drug concentration and a therapeutic effect, determining the clinical and biological impacts of drug and supplement interactions is needed. Although dosing adjustments may be used to counter PK interactions, drug-drug and drug-supplement interactions may still result in PK or PD effects (Asher et al., 2017). A PK interaction may occur, e.g., if a drug has the same mechanism of absorption, distribution, metabolism, or excretion (ADME) as a co-administered supplement, whereby competition at ADME processes can both influence the concentration of the drug or supplement at the site of action (Palleria et al., 2013; Asher et al., 2017; Grogan and Preuss, 2023) and affect the expected actions of the drug (Figure 1A). On the other hand, a PD effect may occur if one drug or supplement directly impacts the mechanism of another drug or supplement, and may alter the clinical efficacy of a drug without any associated changes in drug concentration (Palleria et al., 2013; Asher et al., 2017; Niu et al., 2019) (Figure 1B). With respect to drug-drug interactions, a data mining analysis of the FDA’s AE Reporting System (AERS) for side effect profiles found that two highly prescribed drugs, the lipid-lowering agent pravastatin and the anti-depressant paroxetine, had synergistic effects on blood glucose levels only when taken together (Tatonetti et al., 2011). In a separate analysis of AERS, the co-administration of the diabetes drug rosiglitazone and the incretin mimetic exenatide dramatically decreased myocardial infarctions associated with rosiglitazone alone (Zhao et al., 2013). This study further found 19,133 drug combinations whereby one drug may reduce AEs associated with a second drug (Zhao et al., 2013). Another study that evaluated patients who received triple anti-epileptic drug combinations, found that AEs and seizures occurred more often in patients taking three or four drugs together (Grundmann et al., 2017). Such compelling findings provide evidence of how drug interactions may yield both positive and negative impacts. Examples of known drug-supplement interactions include: goldenseal (*Hydrastis canadensis*) supplements which are recommended not to be administered in combination with the majority of OTC and prescription drugs; and St. John’s wort (*Hypericum perforatum*), which can decrease the efficacy of numerous drugs including warfarin, protease inhibitors, irinotecan, theophylline, and oral contraceptives (Asher et al., 2017). Characterizing and predicting the effects of such interactions will be important for the development of feedback-controlled closed loop TDM solutions to maximize therapeutic benefits.

One strategy that will assist with the management of potential drug and supplement interactions are multiplexed TDM solutions to measure concentrations of multiple targets. ELAs are being developed to measure multiple antibiotics simultaneously (Kling et al., 2016) and mass spectrometry-based methods (e.g.,,LC-MS/MS) have been developed to perform TDM of multiple immunosuppressant drugs (Yang and Wang, 2008; Seger et al., 2009), anti-viral drugs (Conti et al., 2018), antibiotics (Kling et al., 2016; Schuster et al., 2018), anti-depressants (Lindner et al., 2019), anti-psychotic medications (Patteet et al., 2014), and mono-clonal antibodies (Willeman et al., 2019). While these traditional TDM methods are accurate and precise, they suffer from high instrumentation costs, increased turnaround times, and the need for analyses to be performed in clinical laboratories. Although optical-based biosensor solutions for identification of multiple proteins (Spindel and Sapsford, 2014; Rafat et al., 2023) could ultimately be adopted for TDM, continued improvements in biosensors are needed to enable the detection and monitoring of multiple drugs and supplements from the same matrices.

### TDM within N-of-1 trial designs

In the clinical setting, TDM categorizes drug concentrations as sub-therapeutic, therapeutic, supra-therapeutic, or toxic based on statistically determined ranges from clinical trials or in healthy populations (Cooney et al., 2017; Ates et al., 2020), or expert opinion (Cooney et al., 2017). However, such trials did not account for an individual’s unique clinical, genetic, phenotypic, or other, features which may influence TDM measurements and interpretation of data. In other words, although the basic premise of TDM is that a drug’s PK is informative of PD, this does not always hold true (Open Resources for Nursing and Ernstmeyer, 2023), but the use of N-of-1 trials will help to shed light into inter-individual PK and PD variability. Therapeutic ranges may also be modified by electrolyte balance, acid-base balance, age, bacterial resistance, plasma protein binding, or drug interactions (Aronson and Hardman, 1992). It is well known that people treated with drugs such as phenytoin, warfarin, digoxin, and fentanyl, have inter-individual PD variability at a given drug plasma concentration, as well as significant cross-subject differences in steady state plasma drug concentrations (Kang and Lee, 2009; Bahrami et al., 2023), which has also been observed for anti-cancer drugs (Cardoso et al., 2020). Further, studies may not repeat TDM measurements, but the predictive performance of model-informed precision dosing has been found to improve with the addition of longitudinal TDM data (Wicha et al., 2021).

One solution may be evaluating the efficacy and safety of drugs by incorporating TDM into an N-of-1 trial design alongside longitudinal biomonitoring and deep phenotyping of individuals (Lillie et al., 2011; Schork and Goetz, 2017). This design may be used to perform TDM, followed by aggregation of cross-trial data to identify potential sub-populations and TDM trends that may be associated with covariates such as genetic and pharmacogenomic variants or clinical characteristics, including sex, body weight, comorbidities, and other features (Buclin et al., 2020). Since bodily distribution of drugs exhibits both spatial and temporal differences, there may be differences in organ-specific drug kinetics after systemic drug administration (Weiss, 1999; Bian et al., 2021), and an N-of-1 approach will help to better characterize these nuances, as well as cross-subject PK and PD variability (Levy, 1994; Gross, 2001; Kang and Lee, 2009), in order to optimize PK/PD models for TDM. Moreover, longitudinal, and ideally continuous, single subject analyses that incorporate TDM, will help to better define the relationship between drug availability (i.e., dose), therapeutic impact, and physiological functions, while minimizing drug toxicity.

Another complication with TDM is improving drug dose optimization and treatment management in ways that are therapeutically beneficial for the patient. The use of population-based PK data to determine dosing algorithms overlooks numerous factors unique to each person. However, strategies such as incorporating the use of patient-derived organoids to perform drug screening, dose optimization, and treatment holds promise for improving patient outcomes (Bose et al., 2021). Patient-derived organoids capture important patient-specific features, including the patient’s physiology and tissue microenvironments, and is being explored for the treatment of different cancers (Zhou et al., 2021; Shin et al., 2023; Schmäche et al., 2024), including assessing cancer drug resistance (Sun et al., 2024), and digestive disorders (Wang et al., 2022), to name a few. Integrating this approach with patient-focused N-of-1 trials will help to identify, handle, and mitigate issues associated with using population-based data. They can also create opportunities to establish more effective dosing strategies and treatment regimens.

## Future directions

The development of biosensor and TDM technologies creates a valuable opportunity to improve and expedite precision medicine and drug development with the goal of benefitting patients. The continued evolution of nanomaterials, manufacturing and preparation of both electrochemical and optical biosensors, and integration of biosensors into wearables, will have beneficial implications for TDM. The success of TDM relies on the accuracy of measuring drug concentrations in various contexts, and developing sensitive and precise PK/PD models and algorithms, which could in theory be expanded using digital twins and *in silico* clinical trials that are appropriately tailored to each person. The use of TDM as part of N-of-1 trials with longitudinal biomonitoring and deep phenotyping will also enable precision medicine in very appropriate ways. Such studies, in combination with strategies such as patient-derived organoid models, would provide a foundation to improve patient outcomes by optimizing drug dosing and treatment schedules. These studies would also help to identify which individual would benefit from which drugs, and aggregated analyses of N-of-1 studies could identify markers of drug response. Such analyses may also help with, e.g., the identification of molecular PD biomarkers, or drug-specific biomarkers, that reflect biochemical and functional changes in the body that occur in response to a drug (Shipkova and Christians, 2019); the characterization of ADME processes associated with drug-drug and drug-supplement interactions; improving our understanding of human biology (Schork et al., 2023); and validating drug repurposing opportunities and drug-patient matching (Cremers et al., 2016).

While the immense benefits of TDM technologies are apparent, TDM is not without its challenges. However, ongoing efforts across multiple areas will help to pave the way for wider and intelligent adoption of this technology. Implementing continuous TDM will result in the generation of massive amounts of data, which necessitates finding solutions to address data management and data confidentiality. This is exemplified by the use of continuous glucose monitoring, where methods are still evolving to best analyze continuous data (Rodbard, 2016). TDM analyses may also shed light into determining how a drug should be prescribed in order to maximize beneficial clinical outcomes, such that drug candidates that rely on TDM may have lower priority in development pipelines (Buclin et al., 2020). Additionally, TDM may show that patients benefit from lower or fewer doses, or even potentially reveal that certain therapeutics may not be effective. Further, TDM is currently costly, which has limited wider adoption (Buclin et al., 2020; Zhao et al., 2022) such that progress using TDM would benefit from investment from organizations and therapeutic developers. Despite these challenges, the continued development of biosensor technologies and integrating TDM into precision medicine approaches have the potential to significantly improve patient outcomes and positively change the way in which medicine is performed.
